# A multilocus sequence typing scheme of *Pseudomonas putida* for clinical and environmental isolates

**DOI:** 10.1038/s41598-019-50299-6

**Published:** 2019-09-27

**Authors:** Kohei Ogura, Kayo Shimada, Tohru Miyoshi-Akiyama

**Affiliations:** 1 Advanced Health Care Science Research Unit, Institute for Frontier Science Initiative, Kanazawa University, 5-11-80 Kodatsuno, Kanazawa-shi, Ishikawa 920-0942 Japan; 2 Pathogenic Microbe Laboratory, Research Institute, National Center for Global Health and Medicine, 1-21-1 Toyama, Shinjuku-ku, Tokyo 162-8655 Japan

**Keywords:** Prokaryote, Bacterial genes, Bacterial infection

## Abstract

*Pseudomonas putida* is a bacterium commonly found in soils, water and plants. Although *P. putida* group strains are considered to have low virulence, several nosocomial isolates with carbapenem- or multidrug-resistance have recently been reported. In the present study, we developed a multilocus sequence typing (MLST) scheme for *P. putida*. MLST loci and primers were selected and designed using the genomic information of 86 clinical isolates sequenced in this study as well as the sequences of 20 isolates previously reported. The genomes were categorised into 68 sequence types (STs). Significant linkage disequilibrium was detected for the 68 STs, indicating that the *P. putida* isolates are clonal. The MLST tree was similar to the haplotype network tree based on single nucleotide morphisms, demonstrating that our MLST scheme reflects the genetic diversity of *P. putida* group isolated from both clinical and environmental sites.

## Introduction

*Pseudomonas putida*, a rod-shaped gram-negative bacterium, harbours a broad spectrum of metabolic enzymes and is found in edaphic as well as in aquatic environments^1^. Some *P. putida* strains colonises plant roots creating a mutual relationship between the plant and bacteria. This bacterium represents a robust microbial platform for metabolic engineering with biocatalytic activity, thereby conferring it with a high biotechnological value^2^. The complete genome sequences of *P. putida* currently available provides key information on carbohydrate metabolism^3^.

*P. putida* strains have been detected in urine, sputum, blood, wound discharge, peritoneal fluid, cerebrospinal fluid, umbilical swab and other human tissues in hospitals^4–7^. Although the virulence of *P. putida* is lower than that of *Pseudomonas aeruginosa*, *P. putida* infection can be fatal in severely ill or immuno-compromised patients. A recent report has shown that multiple *Pseudomonas* species, including *P. putida*, secrete exolysin‐like toxins and provoke macrophage death^8^. Molina *et al*. reported that clinical isolates, but not environmental isolates, of *P. putida* harbour a set of genes that are involved in survival under oxidative stress conditions and resistance against biocides via amino acid metabolism and toxin/antitoxin systems^9^. Multidrug-resistant and carbapenem-resistant isolates are involved in nosocomial infections. Peter *et al*. reported that 46.1% of these strains isolated from patients with hemato-oncology disorders harboured the metallo-β-lactamase (*blaVIM*) gene^10^. Although quinolone is effective in treating nosocomial infections, including those caused by *Pseudomonas* species, some strains develop quinolone resistance^11^.

Genetic typing allows to distinguish between virulent and non-virulent strains and helps in the biotechnological engineering of *P. putida*. Molina *et al*. reported that clinical and environmental *P. putida* isolates can be grouped into five clades based on their FpvA protein variants, which show high divergence and substantial intra-type variation^12^. Yonezuka *et al*. conducted a phylogenetic analysis based on 16S rRNA analysis, concatenated sequences of nine housekeeping genes and average nucleotide identity (ANI)^13^. They concluded that the analysis based on the concatenated sequences as well as on ANI showed high resolution. However, such phylogenetic analysis requires whole genome sequence data and bioinformatics techniques. Multilocus sequence typing (MLST) provides a universal, portable and precise technique for bacterial typing^14–16^. MLST is available for both whole genome data and Sanger sequencing after PCR. In addition, PubMLST webtool (https://pubmlst.org/) can automatically extract allele profiles and present sequence types (STs). MLST schemes of *Pseudomonas aeruginosa* and *Pseudomonas fluorescens* were reported in 2004 and 2014, respectively^17,18^. Although a multilocus sequence analysis has previously been reported^13^, no MLST scheme has yet been provided for *P. putida*. We developed a MLST scheme using 8 housekeeping genes extracted from whole genome sequences of 86 *P. putida* strains recently isolated from clinical sites in Japan, in addition to the complete genome sequences accessible in the public database.

## Results

### Whole genome sequencing of clinical *P. putida* strains

In order to develop an MLST scheme, about 100 isolates are generally required^15,16^. In the NCBI database, 20 complete genomes were available in 2018. In addition, we sequenced the whole genomes of 86 *P. putida* strains isolated at clinical sites in Japan (Table S1). Among the 20 strains in the NCBI database, 16 strains were isolated from environmental samples, such as soils, plants, and water. The source of the 86 clinical isolates were mainly urine (17strains), eye discharge (16 strains), skin (10 strains), wounds (10 strains), vaginal discharge (8 strains), otorrhea (4 strains), blood (3 strains), and the nasal cavity (3 strains). Among the strains sequenced, PPJ_NCGM_012 (from urine), _78 (from skin), and _92 (from urine) harbored antimicrobial-resistant genes but none carried *blaVIM* conferring β-lactam antibiotic resistance^19^ (Table S2).

### Selection of MLST loci

Using the 106 genomes, we selected loci for MLST. First we chose 17 genes (*acsA, argS, aroE, dnaN, dnaQ, era, gltA, guaA, gyrB, ileS, mutL, nuoC, ppnK, ppsA, recA, rpoB, rpoD* and *trpE*), which are utilized in MLST schemes of other *Pseudomonas* group (*P*. *aeruginosa* and *P. fluorescens*) and a multilocus sequence analysis (MLSA) of *P. putida* group^13,20^. Among the 17 genes, consensus sequences applicable for primers were found in 8 housekeeping genes (*argS*, *gyrB*, *ileS*, *nuoC*, *ppsA*, *recA*, *rpoB* and *rpoD* genes) which encode Arginine–tRNA ligase (ArgS), DNA gyrase subunit B (GyrB), Isoleucine–tRNA ligase (IleS), NADH-quinone oxidoreductase subunit C/D (NuoC), Phosphoenolpyruvate synthase (PpsA), DNA recombination and repair protein (RecA), DNA-directed RNA polymerase subunit beta (RpoB), and RNA polymerase sigma factor (RpoD), respectively. The primers listed in Table 1 were used to confirm amplification by PCR. All PCR amplicons localised at the predicted corresponding sizes in electrophoresis. MLST allele sequences were extracted from sequences of the PCR amplicons.

**Table 1:** Primer sequences and characteristics of MLST loci.

Gene	Forward primer	Reverse primer	Amplicon size	Alleles	S (mean)	N (mean)	n (S + N)	Pairwise comparisons	d_N_	σ d_N_	d_S_
*argS*	ACYCAGTTCGGCATG	TTGAAGCTGTAGTCRCTGGT	845	40	89	274	363	780	0.0172	0.0099	0.3103
*gyrB*	CTGCACCACATGGTNTTCGA	GCATCGGTCATGATGATGATGTTGTGRTA	1368	48	134.9	417.1	552	1128	0.0047	0.003	0.5577
*ileS*	CCSTACAAGACCATG	CGCCAGCARTGCAT	762	48	144.1	389.9	534	1128	0.0354	0.026	0.3198
*nuoC*	GCCARCTGGTACGA	GCCTTGTACGGGCCTKC	1068	47	141.7	350.3	492	1081	0.0569	0.0215	0.0566
*ppsA*	TTCATCATCAACCGCAYGAT	AGGWAGAACCAGGT	808	47	122.1	375.9	498	1081	0.0673	0.0289	0.0522
*recA*	ATGGACGACAACAAGAA	ACCTTGTTYTTGACGAT	733	48	143.3	402.7	546	1128	0.1009	0.0363	0.0159
*rpoB*	CGTATCCGCAAGGACTTTAGCAAGTTGCCGGACGT	CGYTCGGTACCGTTGAT	409	18	78.3	221.7	300	153	0.0185	0.0099	0.0146
*rpoD*	ATGTCCGGAAAAGCGCAACAGCARTCTCG	CGGTTGGTGTACTTYTTGGCGAT	1190	48	125.3	363.7	489	1128	0.1034	0.0359	0.281

### Development of a MLST scheme for *P. putida*

Table 1 shows the ratios of substitutions at non-silent (non-synonymous, change of amino acid) sites (dN) to those at silent (synonymous, no change of amino acid) sites (dS). The dN/dS ratios were extremely high (dN/dS > 1) in four genes (*nuoC*, *ppsA*, *recA* and *rpoB*), indicating a positive selection in the four housekeeping proteins. To test for positive selection of codons, we used the site-model analysis function of CodeML program, which is a part of the PAML software package^21^. No significant selection of codons was detected. Moreover, no deviation from random evolution was detected among any of the populations following neutrality test using Tajima’s D statistic, Fu’s D and F statistics, or Ramos-Onsins & Rozas’ R2 (Table 1). Our MLST scheme showed that 106 *P. putida* strains were categorised into 68 sequence types (STs) (Table S1). The MLST data have been deposited in the PubMLST database, available for public analysis. The number of unique alleles varied from 18 (*rpoB*) to 48 (*gyrB*, *ileS*, *recA* and *rpoD*) (Table S3).

### Linkage disequilibrium

To detect the non-random association of alleles at different loci, an index of association (I_A_) was calculated. Significant linkage disequilibrium (*P* = 0.0) was detected for the 68 STs with I_A_ = 2.94 in the classical (Maynard Smith) method and I^S^_A_ = 0.42 in the standardised (Haubold) method. The significant linkage disequilibrium revealed close associations among the eight housekeeping genes. In addition, these close associations among the *P. putida* strains indicated that the strains used in this MLST scheme are clonal.

### Tree of the MLST scheme based on unweighted pair group method with arithmetic mean

**Figure 1:**
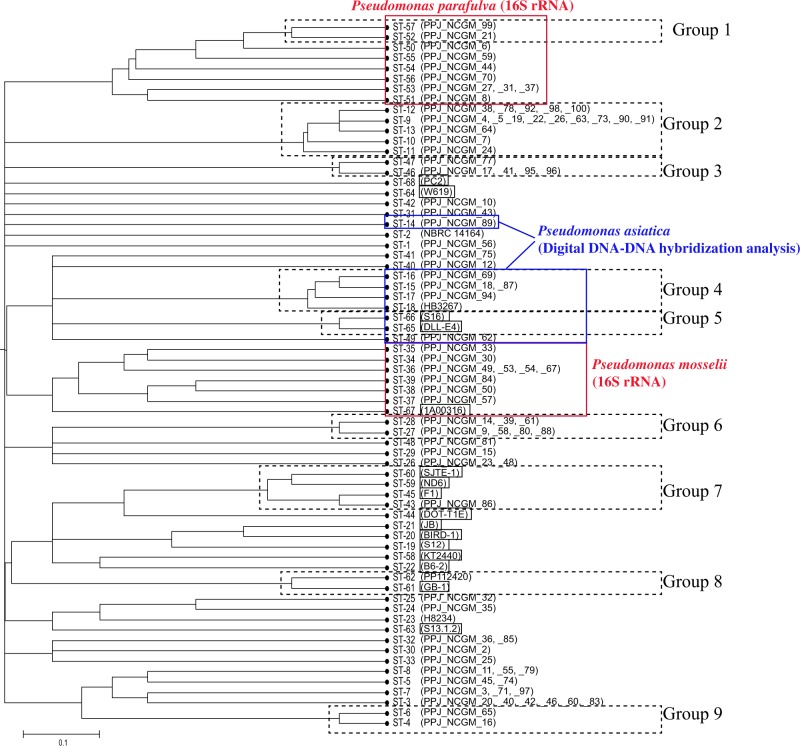
UPGMA tree of 68 sequence types (STs). The UPGMA tree was prepared using 68 STs in START2 programme. Squares indicate environmental isolates. Dash lines indicate clonal complex categorised by the eBURST programme. Red box shows result of 16S rRNA sequence. *P. asiatica*, which was detected by digital DDH analysis, are shown in blue box and line.

Figure 1 shows an unweighted pair group method with arithmetic mean (UPGMA) tree, which was constructed from pairwise differences in the allelic profiles of the 68 STs. The analysis of the clonal complex showed that one group consisted of ST-9, -10, -11, -12 and -13. Although some non-clinical isolates (DOT-T1E, JB, BIRD-1, S12, KT2440 and B6-2) were relatively close, they were not categorised into the same clonal complex.

### Comparison of the MLST scheme with SNP-based distance tree

**Figure 2:**
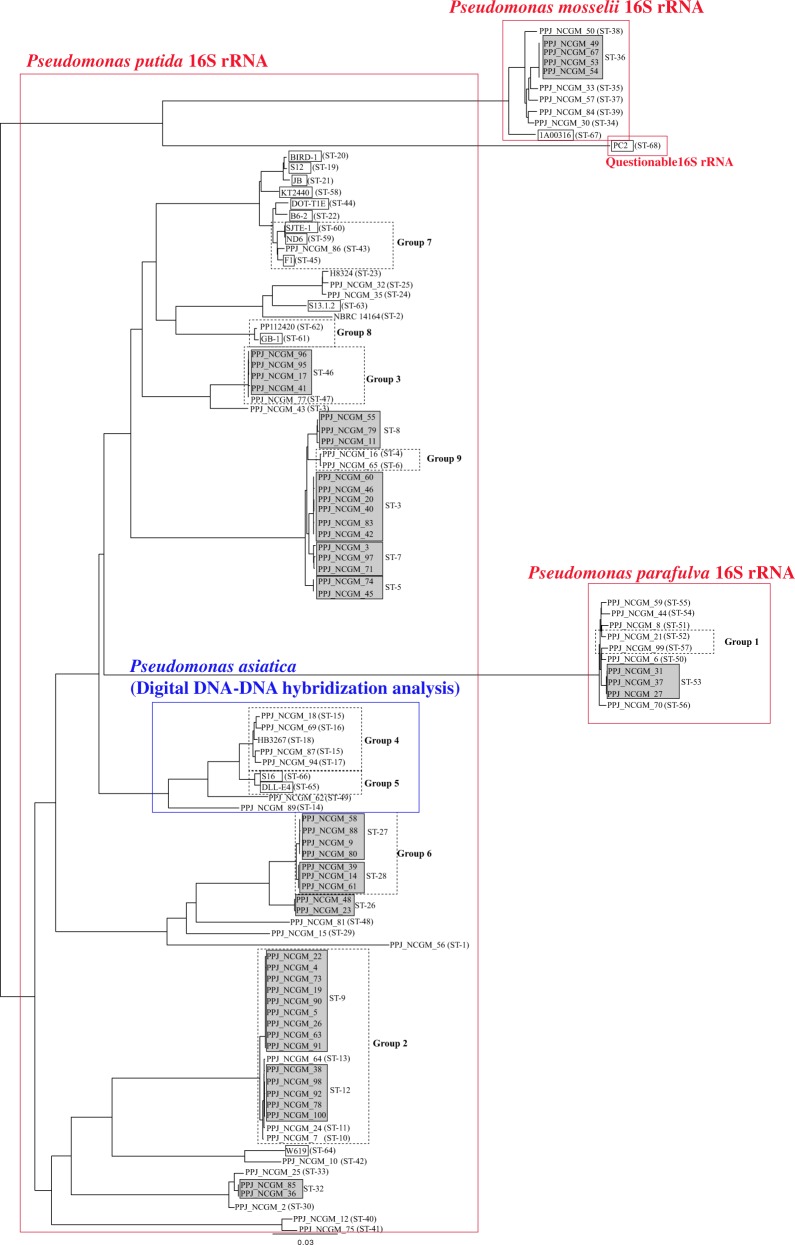
Analysis of concatenated SNPs of 106 strains. The SNP-based distance tree was prepared using 7,194 concatenated SNPs. The haplotype network tree model was prepared using SNiPlay. Trees were visualised using FigTree. Grey and dashed boxes indicate identical STs and clonal complex groups. Red and blue boxes show results of 16S rRNA sequence and digital DDH analysis, respectively.

To evaluate the MLST scheme, we prepared a haplotype network tree based on 7,194 single nucleotide polymorphisms (SNPs) extracted from the total 106 whole genome sequences included in the present study (Fig. 2). Strains belonging to identical STs and identical clonal complex groups were close to each other, which was consistent with the MLST tree. Therefore, the MLST scheme reflects the whole genome profiles and is applicable for the characterisation of *P. putida* isolates.

### 16S rRNA sequences analysis

The 20 complete genomes were registered as *P. putida* genomes. Our 86 strains were judged as *P. putida* by MALDI-TOF MS. 16S rRNA sequence alignment, showed that some of strains were *P. mosselii* and *P. parafulva*, both of which belonged to the *P. putida* group. As shown in Figs 1 and 2, *P. mosselii* and *P. parafulva* strains were closely located in the MLST and SNP trees.

### DNA-DNA distance analysis

To calculate the genetic distance of the 106 strains, digital DNA-DNA hybridization (DDH) was conducted using the KT2440 strain as a query. Interestingly, only 9 strains were estimated to be the same species as the KT2440 strain (Table S1, right end). Further DDH analysis revealed that the 9 strains were *P. asiatica*, whose 16S rRNA sequence was identical to that of *P. putida*. While in the SNP tree (Fig. 2), the 9 *P. asiatica* strains were closely located, in the MLST tree (Fig. 1), one ST (ST-14) branched away from 7 of the STs.

## Discussion

Based on the 16S rRNA sequence, *Pseudomonas* species was categorized into 5 groups; the *P. aeruginosa* group, the *P. chlororaphis* group, the *P. fluorescen*s group, the *P. pertucinogena* group, and the *P. putida* group^22^. We found that this MLST scheme was applicable for *P. putida*, *P. mosselii*, and *P. parafulva*. Recently Tohya *et al*. reported that *P. asiatica*, newly split from *P. putida* based on average nucleotide identity and digital DDH and was phylogenetically close to *Pseudomonas monteilii* and *P. putida*^23^. In this MLST scheme, most of the *P. asiatica* strains were located close to each other, while one strain was not (Fig. 1). This result indicates that some of *P. asiatica* strains could be detected by our MLST scheme.

Yonezuka *et al*. reported that a phylogenetic trees based on 16S rRNA sequences was inadequate and that multi-locus sequence analysis (MLSA) based on 9 concatenated housekeeping genes (*argS-dnaN-dnaQ-era-gltA-gyrB-ppnK-rpoB-rpoD*) improved the resolution of the phylogenetic tree^13^. Consistent with the report, our MLST scheme yielded approximately the same results as the SNP analysis of the whole genomic sequence, while we utilized some different housekeeping genes because some genes lacked consensus sequences for PCR primers (Table 1).

Antibiotic-resistant strains in the *P. putida* group have been increasingly isolated from clinical sites^10,24^. Because none of the 106 strains possessed the *blaVIM* gene, distribution of the genes was not indicated in our MLST scheme.

In conclusion, we developed an MLST scheme for *P. putida* group strains using 8 housekeeping genes. This scheme was applicable to both clinical and environmental isolates.

## Methods

### DNA of bacterial strains

Overall, 86 strains, isolated from clinical sites in Japan in 2017, were judges as P. putida by MALDI-TOF MS (Vitek MS, bioMérieux). The *P. putida* strains were cultured in brain heart infusion media (Becton Dickinson) at 37 °C overnight. Genomic DNA was extracted using DNeasy Blood & Tissue Kit (QIAGEN), according to the manufacturer’s instructions.

### Determination of whole genome sequences

Sequencing libraries were prepared using the Next Ultra II DNA library prep kit for Illumina (New England Biolabs) and subjected to HiSeq X platform (Illumina). The obtained 151 bp paired-end reads were *de novo* assembled to contigs using CLC Genomics Workbench (version 11.0.1, QIAGEN) after trimming depending on the base quality (quality score limit = 0.05)—reads with > 2 ambiguous nucleotides and those <15 bp in length were removed.

### Antimicrobial genes

Drug resistant genes were detected using BLAST against the AMR database of CLC Genomics Workbench. The *blaVIM* genes were retrieved from NCBI database and used for BLAST.

### PCR conditions and amplicon sequencing

According to previous reports^13,17,18^, we selected *acsA, argS, aroE, dnaN, dnaQ, era, gltA, guaA, gyrB, ileS, mutL, nuoC, ppnK, ppsA, recA, rpoB, rpoD* and *trpE* genes as the candidates of MLST loci. These were extracted from the genome sequences of the 86 isolates and from the 20 genome sequences available from the NCBI website by CLC Genomics Workbench, and the sequences were aligned using the MAFFT program^25^. In *argS*, *gyrB*, *ileS*, *nuoC*, *ppsA*, *recA*, *rpoB* and *rpoD* genes, conserved sequences for primers were ‘manually’ identified using the Genetyx software (Genetyx Corporation, Japan; Table 1). PCR amplification thermal cycles consisted of 3 min at 98 °C, 30 cycles at 98 °C for 20 sec, at 45 °C for 20 sec and at 72 °C for 1 min, with a final extension at 72 °C for 5 min using the TaKaRa LA Taq DNA Polymerase (Takara Bio) in the Veriti Thermal Cycler (ThermoFisher Scientific). PCR amplicons were treated with ExoSAP-IT Express PCR Cleanup Reagents (ThermoFisher Scientific) and sequenced using the primers listed in Table 1 and the BigDye Terminator v3.1 Cycle Sequencing Kit (ThermoFisher Scientific) on an ABI 3730xl (ThermoFisher Scientific).

### Sequence typing

Allele sequences of the 8 housekeeping genes from the 86 isolates and the 20 sequences available in the database were ‘manually’ extracted using the Genetyx software and uploaded to PubMLST website^20^. I_A_ values were calculated using the START2 software^26^. The UPGMA tree was prepared in the START2 program^26^. STs were grouped using the BURST program^27^. Tajima’s D statistic, Fu’s F and D statistic and Ramos-Onsins & Rozas’ R2 were tested using the DnaSP 6 software^28–30^.

### Calculation of dN/dS ratio

dN/dS substitution ratio of the allelic genes were calculated using the START2 program^26^. For further analysis of the dN/dS ratio, positive selection (dN/dS > 1) of the allelic genes were examined using the CodeML program in the PAML software package^21^. Codon sequences were manually extracted using Genetyx software. Head and tail nucleotides that do not constitute codons were removed. Moreover, stop codons and sequences after the codons were removed. Phlyip 3.0 format files and Neighbor-Joining tree were prepared in MEGA5 software^31^. Site models were tested for the eight housekeeping genes.

### SNP analysis

SNPs were extracted by the Parsnp tool using KT2440 as a reference after removing the phage regions predicted by the PHAST tool^32,33^. Following the conversion of a VCF file using VCFtools^34^, SNP-based distance tree was constructed using SNiPlay^35^ web-tool. The tree was visualised using FigTree (http://tree.bio.ed.ac.uk/software/figtree/).

### 16S rRNA analysis and digital DNA-DNA hybridization

Sequences of 16S rRNA were extracted from the genome sequences and aligned with NCBI 16S database using commercial software CLC Genomics Workbench. Digital DNA-DNA hybridization was conducted using Genome-to-Genome Distance Calculator 2.1 Webtool^36^.

## Supplementary information

Supplemenrary Materials

## Data Availability

The raw reads data have been registered with DDBJ as Accession Number DRA007569. The MLST scheme is available at PubMLST website (https://pubmlst.org/pputida/).
