# Supplementary material for: A multilocus sequence typing scheme of Pseudomonas putida for clinical and environmental isolates
Source: Sci Rep. 2019 Sep 27;9:13980. doi: 10.1038/s41598-019-50299-6 (PMC6765009; doi:10.1038/s41598-019-50299-6)
Supplement: Supplementary file 1 — Supplemenrary Materials [file 41598_2019_50299_MOESM1_ESM.pdf]

# **Supplementary information:** A multilocus sequence typing scheme of *Pseudomonas putida* for clinical and environmental isolates

Kohei Ogura[1*], Kayo Shimada[2], and Tohru Miyoshi-Akiyama[2]

1. Advanced Health Care Science Research Unit, Institute for Frontier Science Initiative, Kanazawa University, 5-11-80 Kodatsuno, Kanazawa-shi, Ishikawa 920-0942, Japan
2. Pathogenic Microbe Laboratory, Research Institute, National Center for Global Health and Medicine, 1-21-1 Toyama, Shinjuku-ku, Tokyo 162-8655, Japan

[*]ogura@staff.kanazawa-u.ac.jp

## Table S1. Strains used in this study

| | | | | | | | Accession numbers in pubMLST database | | | | | | | | | |
|---|---|---|---|---|---|---|---|---|---|---|---|---|---|---|---|---|
| | | | | | | | | genes | | | | | | | | Digital DDH (%) |
| isolate | Reference | country | year | source | detailed_source | disease | **ST** | *argS* | *gyrB* | *ileS* | *nuoC* | *ppsA* | *recA* | *rpoB* | *rpoD* | (Query: KT2440) |
| ND6 | NC_017986.1 | China | | environmental | industrial wastewater | | **59** | 38 | 14 | 8 | 4 | 10 | 12 | 1 | 6 | 74.1 |
| KT2440 | NC_002947.4 | Japan | | environmental | | | **58** | 37 | 44 | 3 | 45 | 2 | 1 | 1 | 1 | 100.0 |
| S12 | NZ_CP009974.1 | The Netherlands | 1989 | environmental | soil | | **19** | 12 | 45 | 6 | 3 | 19 | 4 | 1 | 2 | 76.1 |
| BIRD-1 | NC_017530.1 | Unknown | | environmental | soil | | **20** | 12 | 46 | 7 | 3 | 16 | 5 | 1 | 2 | 74.5 |
| JB | NZ_CP016212.1 | Czech Republic | 2000 | environmental | | | **21** | 12 | 46 | 7 | 3 | 21 | 6 | 1 | 3 | 75.2 |
| DOT-T1E | CP003734.1 | Unknown | | environmental | | | **44** | 31 | 47 | 10 | 4 | 8 | 7 | 1 | 8 | 73.1 |
| B6-2 | NZ_CP015202.1 | Unknown | | environmental | | | **22** | 13 | 13 | 3 | 44 | 3 | 11 | 1 | 9 | 76.0 |
| SJTE-1 | NZ_CP015876.1 | China | | environmental | soil | | **60** | 38 | 14 | 9 | 4 | 9 | 12 | 1 | 6 | 76.1 |
| F1 | NC_009512.1 | Unknown | | environmental | | | **45** | 31 | 14 | 8 | 4 | 13 | 12 | 1 | 6 | 75.6 |
| GB-1 | NC_010322.1 | Unknown | | environmental | | | **61** | 39 | 48 | 8 | 46 | 11 | 14 | 4 | 14 | 61.4 |
| S13.1.2 | NZ_CP010979.1 | France | 2008 | environmental | soil | | **63** | 40 | 49 | 28 | 47 | 12 | 15 | 12 | 25 | 57.2 |
| PP112420 | NZ_CP017073.1 | China | 2011 | clinical | urine | urinary tract infection | **62** | 39 | 48 | 15 | 48 | 11 | 14 | 4 | 14 | 61.6 |
| H8234 | NC_021491.1 | France | | clinical | blood | septicaemia | **23** | 14 | 10 | 33 | 9 | 32 | 17 | 12 | 23 | 54.8 |
| NBRC 14164 | NC_021505.1 | Unknown | | clinical | | | **2** | 3 | 15 | 29 | 5 | 17 | 20 | 10 | 21 | 60.2 |
| W619 | NC_010501.1 | Unknown | | environmental | soil | | **64** | 41 | 50 | 42 | 49 | 48 | 29 | 18 | 48 | 48.5 |
| HB3267 (PC9) | NC_019905.1 | France | | clinical | | | **18** | 11 | 24 | 20 | 15 | 39 | 33 | 7 | 28 | 59.0 |
| DLL-E4 | NZ_CP007620.1 | China | 2000 | environmental | soil | | **65** | 42 | 51 | 21 | 50 | 23 | 34 | 7 | 31 | 58.8 |
| S16 | NC_015733.1 | Unknown | | environmental | | | **66** | 42 | 51 | 21 | 50 | 46 | 34 | 7 | 31 | 59.4 |
| 1A00316 | NZ_CP014343.1 | Unknown | 2013 | environmental | soil | | **67** | 43 | 52 | 60 | 51 | 44 | 45 | 23 | 61 | 41.2 |
| PC2 | NZ_CP011789.1 | China | | environmental | seed | | **68** | 44 | 53 | 68 | 52 | 53 | 54 | 26 | 67 | 37.6 |
| NCGM_2 | This study | Japan | 2017 | clinical | sputum | pneumonia | **30** | 20 | 42 | 36 | 12 | 37 | 52 | 19 | 51 | 46.5 |
| NCGM_3 | | Japan | 2017 | clinical | pharynx | other | **7** | 6 | 6 | 50 | 26 | 33 | 59 | 13 | 37 | 50.6 |
| NCGM_4 | | Japan | 2017 | clinical | eye discharge | other | **9** | 8 | 11 | 51 | 31 | 51 | 55 | 20 | 52 | 43.1 |
| NCGM_5 | | Japan | 2017 | clinical | eye discharge | other | **9** | 8 | 11 | 51 | 31 | 51 | 55 | 20 | 52 | 43.3 |
| NCGM_6 | | Japan | 2017 | clinical | eye discharge | other | **50** | 36 | 38 | 71 | 38 | 56 | 64 | 15 | 59 | 36.2 |
| NCGM_7 | | Japan | 2017 | clinical | sperm | other | **10** | 8 | 11 | 52 | 31 | 51 | 55 | 20 | 53 | 43.3 |
| NCGM_8 | | Japan | 2017 | clinical | eye discharge | other | **51** | 36 | 36 | 74 | 40 | 59 | 65 | 15 | 59 | 36.9 |
| NCGM_9 | | Japan | 2017 | clinical | foreskin | other | **27** | 18 | 26 | 46 | 22 | 41 | 24 | 21 | 41 | 47.3 |
| NCGM_10 | | Japan | 2017 | clinical | otorrhea | other | **42** | 30 | 32 | 41 | 30 | 50 | 31 | 17 | 46 | 48.7 |
| NCGM_11 | | Japan | 2017 | clinical | urine | urinary tract infection | **8** | 7 | 4 | 49 | 23 | 33 | 59 | 13 | 38 | 52.5 |
| NCGM_12 | | Japan | 2017 | clinical | urine | urinary tract infection | **40** | 28 | 17 | 39 | 28 | 31 | 27 | 7 | 55 | 49.8 |
| NCGM_14 | | Japan | 2017 | clinical | wound | wound infection | **28** | 18 | 26 | 46 | 22 | 41 | 24 | 21 | 42 | 50.4 |
| NCGM_15 | | Japan | 2017 | clinical | nasal cavity | other | **29** | 19 | 31 | 44 | 20 | 42 | 26 | 21 | 40 | 49.9 |
| NCGM_16 | | Japan | 2017 | clinical | vaginal discharge | other | **4** | 5 | 2 | 48 | 24 | 34 | 59 | 13 | 34 | 51.4 |
| NCGM_17 | | Japan | 2017 | clinical | skin | other | **46** | 32 | 20 | 16 | 14 | 30 | 40 | 6 | 17 | 57.0 |
| NCGM_18 | | Japan | 2017 | clinical | urine | urinary tract infection | **15** | 11 | 24 | 20 | 15 | 20 | 33 | 7 | 28 | 58.2 |
| NCGM_19 | | Japan | 2017 | clinical | cornea | other | **9** | 8 | 11 | 51 | 31 | 51 | 55 | 20 | 52 | 41.5 |
| NCGM_20 | | Japan | 2017 | clinical | skin | other | **3** | 4 | 5 | 50 | 25 | 33 | 57 | 13 | 35 | 50.3 |
| NCGM_21 | | Japan | 2017 | clinical | urine | wound infection | **52** | 36 | 35 | 71 | 38 | 58 | 64 | 15 | 58 | 37.5 |
| NCGM_22 | | Japan | 2017 | clinical | vaginal discharge | other | **9** | 8 | 11 | 51 | 31 | 51 | 55 | 20 | 52 | 42.8 |
| NCGM_23 | | Japan | 2017 | clinical | pharynx | other | **26** | 17 | 25 | 45 | 21 | 40 | 25 | 21 | 43 | 50.0 |
| NCGM_24 | | Japan | 2017 | clinical | skin | other | **11** | 8 | 11 | 52 | 31 | 51 | 56 | 20 | 52 | 42.6 |
| NCGM_25 | | Japan | 2017 | clinical | urine | urinary tract infection | **33** | 24 | 43 | 37 | 10 | 38 | 51 | 19 | 49 | 47.1 |
| NCGM_26 | | Japan | 2017 | clinical | urine | urinary tract infection | **9** | 8 | 11 | 51 | 31 | 51 | 55 | 20 | 52 | 41.5 |
| NCGM_27 | | Japan | 2017 | clinical | wound | wound infection | **53** | 36 | 37 | 75 | 41 | 60 | 67 | 15 | 59 | 35.7 |
| NCGM_30 | | Japan | 2017 | clinical | blood | septicaemia | **34** | 25 | 29 | 58 | 35 | 46 | 43 | 23 | 62 | 40.2 |
| NCGM_31 | | Japan | 2017 | clinical | sputum | pneumonia | **53** | 36 | 37 | 75 | 41 | 60 | 67 | 15 | 59 | 36.0 |
| NCGM_32 | | Japan | 2017 | clinical | nasal cavity | other | **25** | 16 | 8 | 33 | 7 | 13 | 16 | 12 | 24 | 56.2 |

| | | | | | | | | | | | | | | | |
|---|---|---|---|---|---|---|---|---|---|---|---|---|---|---|---|
| NCGM_35 | Japan | 2017 | clinical | wound | wound infection | **24** | 15 | 9 | 33 | 8 | 14 | 16 | 12 | 24 | 40.0 |
| NCGM_33 | Japan | 2017 | clinical | eye discharge | other | **35** | 25 | 30 | 55 | 32 | 46 | 49 | 23 | 63 | 58.5 |
| NCGM_36 | Japan | 2017 | clinical | digestive organ | other | **32** | 23 | 41 | 35 | 11 | 36 | 50 | 19 | 50 | 45.1 |
| NCGM_37 | Japan | 2017 | clinical | urine | urinary tract infection | **53** | 36 | 37 | 75 | 41 | 60 | 67 | 15 | 59 | 36.1 |
| NCGM_38 | Japan | 2017 | clinical | urine | urinary tract infection | **12** | 8 | 11 | 52 | 31 | 51 | 55 | 20 | 52 | 44.3 |
| NCGM_39 | Japan | 2017 | clinical | pharynx | other | **28** | 18 | 26 | 46 | 22 | 41 | 24 | 21 | 42 | 50.4 |
| NCGM_40 | Japan | 2017 | clinical | skin | other | **3** | 4 | 5 | 50 | 25 | 33 | 57 | 13 | 35 | 50.2 |
| NCGM_41 | Japan | 2017 | clinical | eye discharge | other | **46** | 32 | 20 | 16 | 14 | 30 | 40 | 6 | 17 | 56.9 |
| NCGM_42 | Japan | 2017 | clinical | pharynx | other | **3** | 4 | 5 | 50 | 25 | 33 | 57 | 13 | 35 | 50.8 |
| NCGM_43 | Japan | 2017 | clinical | eye discharge | other | **31** | 21 | 18 | 18 | 13 | 28 | 41 | 5 | 16 | 61.6 |
| NCGM_44 | Japan | 2017 | clinical | urine | urinary tract infection | **54** | 36 | 33 | 72 | 42 | 56 | 63 | 15 | 60 | 36.3 |
| NCGM_45 | Japan | 2017 | clinical | urine | urinary tract infection | **5** | 5 | 3 | 47 | 23 | 33 | 58 | 13 | 36 | 51.4 |
| NCGM_46 | Japan | 2017 | clinical | vaginal discharge | other | **3** | 4 | 5 | 50 | 25 | 33 | 57 | 13 | 35 | 53.3 |
| NCGM_48 | Japan | 2017 | clinical | wound | wound infection | **26** | 17 | 25 | 45 | 21 | 40 | 25 | 21 | 43 | 49.5 |
| NCGM_49 | Japan | 2017 | clinical | skin | other | **36** | 25 | 27 | 56 | 36 | 43 | 48 | 23 | 62 | 39.1 |
| NCGM_50 | Japan | 2017 | clinical | eye discharge | other | **38** | 26 | 28 | 57 | 33 | 45 | 44 | 23 | 64 | 41.0 |
| NCGM_53 | Japan | 2017 | clinical | otorrhea | other | **36** | 25 | 27 | 56 | 36 | 43 | 48 | 23 | 62 | 39.1 |
| NCGM_54 | Japan | 2017 | clinical | skin | other | **36** | 25 | 27 | 56 | 36 | 43 | 48 | 23 | 62 | 39.2 |
| NCGM_55 | Japan | 2017 | clinical | urine | urinary tract infection | **8** | 7 | 4 | 49 | 23 | 33 | 59 | 13 | 38 | 52.1 |
| NCGM_56 | Japan | 2017 | clinical | vaginal discharge | other | **1** | 1 | 7 | 61 | 37 | 62 | 61 | 22 | 44 | 40.5 |
| NCGM_57 | Japan | 2017 | clinical | wound | wound infection | **37** | 25 | 28 | 54 | 34 | 46 | 46 | 23 | 64 | 40.4 |
| NCGM_58 | Japan | 2017 | clinical | wound | wound infection | **27** | 18 | 26 | 46 | 22 | 41 | 24 | 21 | 41 | 48.1 |
| NCGM_59 | Japan | 2017 | clinical | wound | wound infection | **55** | 36 | 34 | 73 | 38 | 57 | 63 | 15 | 58 | 36.1 |
| NCGM_60 | Japan | 2017 | clinical | skin | other | **3** | 4 | 5 | 50 | 25 | 33 | 57 | 13 | 35 | 50.0 |
| NCGM_61 | Japan | 2017 | clinical | eye discharge | other | **28** | 18 | 26 | 46 | 22 | 41 | 24 | 21 | 42 | 47.8 |
| NCGM_62 | Japan | 2017 | clinical | otorrhea | other | **49** | 35 | 23 | 26 | 17 | 25 | 38 | 7 | 27 | 56.8 |
| NCGM_63 | Japan | 2017 | clinical | skin | other | **9** | 8 | 11 | 51 | 31 | 51 | 55 | 20 | 52 | 43.6 |
| NCGM_64 | Japan | 2017 | clinical | catheter | other | **13** | 8 | 11 | 53 | 31 | 51 | 55 | 20 | 52 | 42.9 |
| NCGM_65 | Japan | 2017 | clinical | catheter | other | **6** | 5 | 2 | 48 | 24 | 34 | 57 | 13 | 34 | 53.6 |
| NCGM_67 | Japan | 2017 | clinical | skin | other | **36** | 25 | 27 | 56 | 36 | 43 | 48 | 23 | 62 | 39.1 |
| NCGM_69 | Japan | 2017 | clinical | sputum | urinary tract infection | **16** | 11 | 24 | 20 | 16 | 20 | 33 | 7 | 28 | 56.7 |
| NCGM_70 | Japan | 2017 | clinical | eye discharge | other | **56** | 36 | 37 | 71 | 39 | 61 | 66 | 15 | 57 | 37.3 |
| NCGM_71 | Japan | 2017 | clinical | wound | wound infection | **7** | 6 | 6 | 50 | 26 | 33 | 59 | 13 | 37 | 51.2 |
| NCGM_73 | Japan | 2017 | clinical | wound | wound infection | **9** | 8 | 11 | 51 | 31 | 51 | 55 | 20 | 52 | 42.1 |
| NCGM_74 | Japan | 2017 | clinical | urine | other | **5** | 5 | 3 | 47 | 23 | 33 | 58 | 13 | 36 | 51.3 |
| NCGM_75 | Japan | 2017 | clinical | blood | other | **41** | 29 | 21 | 38 | 29 | 32 | 28 | 7 | 54 | 49.6 |
| NCGM_77 | Japan | 2017 | clinical | eye discharge | other | **47** | 32 | 20 | 16 | 14 | 30 | 40 | 6 | 18 | 57.3 |
| NCGM_78 | Japan | 2017 | clinical | skin | other | **12** | 8 | 11 | 52 | 31 | 51 | 55 | 20 | 52 | 43.7 |
| NCGM_79 | Japan | 2017 | clinical | wound | wound infection | **8** | 7 | 4 | 49 | 23 | 33 | 59 | 13 | 38 | 56.5 |
| NCGM_80 | Japan | 2017 | clinical | urine | urinary tract infection | **27** | 18 | 26 | 46 | 22 | 41 | 24 | 21 | 41 | 47.5 |
| NCGM_81 | Japan | 2017 | clinical | blood | septicaemia | **48** | 33 | 39 | 82 | 19 | 39 | 23 | 21 | 39 | 49.5 |
| NCGM_83 | Japan | 2017 | clinical | nasal cavity | other | **3** | 4 | 5 | 50 | 25 | 33 | 57 | 13 | 35 | 52.2 |
| NCGM_84 | Japan | 2017 | clinical | eye discharge | other | **39** | 27 | 28 | 59 | 33 | 47 | 47 | 23 | 64 | 40.7 |
| NCGM_85 | Japan | 2017 | clinical | vaginal discharge | other | **32** | 23 | 41 | 35 | 11 | 36 | 50 | 19 | 50 | 46.0 |
| NCGM_86 | Japan | 2017 | clinical | CAPD tube | other | **43** | 31 | 14 | 8 | 4 | 4 | 12 | 1 | 6 | 74.2 |
| NCGM_87 | Japan | 2017 | clinical | urine | urinary tract infection | **15** | 11 | 24 | 20 | 15 | 20 | 33 | 7 | 28 | 56.4 |
| NCGM_88 | Japan | 2017 | clinical | vaginal discharge | other | **27** | 18 | 26 | 46 | 22 | 41 | 24 | 21 | 41 | 47.9 |
| NCGM_89 | Japan | 2017 | clinical | urine | urinary tract infection | **14** | 10 | 22 | 24 | 18 | 27 | 32 | 8 | 33 | 57.5 |
| NCGM_90 | Japan | 2017 | clinical | eye discharge | other | **9** | 8 | 11 | 51 | 31 | 51 | 55 | 20 | 52 | 41.5 |
| NCGM_91 | Japan | 2017 | clinical | eye discharge | other | **9** | 8 | 11 | 51 | 31 | 51 | 55 | 20 | 52 | 43.4 |
| NCGM_92 | Japan | 2017 | clinical | urine | urinary tract infection | **12** | 8 | 11 | 52 | 31 | 51 | 55 | 20 | 52 | 43.8 |
| NCGM_94 | Japan | 2017 | clinical | vaginal discharge | other | **17** | 11 | 24 | 20 | 15 | 20 | 33 | 7 | 29 | 55.1 |

| | | | | | | | | | | | | | | | |
|---|---|---|---|---|---|---|---|---|---|---|---|---|---|---|---|
| NCGM_95 | Japan | 2017 | clinical | otorrhea | other | **46** | 32 | 20 | 16 | 14 | 30 | 40 | 6 | 17 | 54.3 |
| NCGM_96 | Japan | 2017 | clinical | eye discharge | other | **46** | 32 | 20 | 16 | 14 | 30 | 40 | 6 | 17 | 55.3 |
| NCGM_97 | Japan | 2017 | clinical | urine | urinary tract infection | **7** | 6 | 6 | 50 | 26 | 33 | 59 | 13 | 37 | 50.9 |
| NCGM_98 | Japan | 2017 | clinical | vaginal discharge | other | **12** | 8 | 11 | 52 | 31 | 51 | 55 | 20 | 52 | 44.2 |
| NCGM_99 | Japan | 2017 | clinical | pituitary gland | meningitis | **57** | 36 | 33 | 71 | 38 | 57 | 64 | 15 | 58 | 35.9 |
| NCGM_100 | Japan | 2017 | clinical | eye discharge | other | **12** | 8 | 11 | 52 | 31 | 51 | 55 | 20 | 52 | 45.4 |

Alelle sequences are available in pubMLST webite (https://pubmlst.org/bigsdb?db=pubmlst_pputida_seqdef)

**Table S2.** ***Pseudomonas putida*** **strains harbouring drug resistant genes.**

| Isolate | Gene | Function |
|---|---|---|
| PP112420 | *blaVIM* | metallo-β-lactamase |
| | *aac(6')-Ib-cr* | aminoglycoside |
| | *aac(6')-Ib3* | aminoglycoside |
| | *qnrVC6* | quinolone |
| | *sul1* | sulphonamide |
| HB3267 | *blaVIM* | metallo-β-lactamase |
| | *aadA1* | Aminoglycoside resistance |
| | *strA* | Aminoglycoside resistance |
| | *aph(3')-Ia* | Aminoglycoside resistance |
| | *aac(6')-Ib-cr* | Fluoroquinolone and aminoglycoside resistance |
| | *ant(2'')-Ia* | Aminoglycoside resistance |
| | *aac(6')-Ib3* | Aminoglycoside resistance |
| | *aph(3'')-Ib* | Aminoglycoside resistance |
| | *aph(6)-Id* | Aminoglycoside resistance |
| | *cmlA1* | Phenicol resistance |
| | *sul2* | Sulphonamide resistance |
| | *sul1* | Sulphonamide resistance |
| | *tet(A)* | Tetracycline resistance |
| H8234 | *strA* | Aminoglycoside resistance |
| | *aph(3'')-Ib* | Aminoglycoside resistance |
| | *aph(6)-Id* | Aminoglycoside resistance |
| DLL-E4 | *sul1* | Sulphonamide resistance |
| PPJ_NCGM_012 | *strA* | Aminoglycoside resistance |
| | *aph(3'')-Ib* | Aminoglycoside resistance |
| | *aph(6)-Id* | Aminoglycoside resistance |
| PPJ_NCGM_078 | *sul1* | Sulphonamide resistance |
| PPJ_NCGM_092 | *aac(6')-Iae* | Aminoglycoside resistance |
| | *blaCMY-8* | β-lactam resistance |
| | *ere(A)* | Macrolide resistance |
| | *sul1* | Sulphonamide resistance |

**Table S3. Allele frequencies of the MLST scheme for *Pseudomonas putida***

| Allele | *argS* | *gyrB* | *ileS* | *nuoC* | *ppsA* | *recA* | *rpoB* | *rpoD* |
|---|---|---|---|---|---|---|---|---|
| **1** | 1 | - | - | - | - | 1 | 10 | 1 |
| **2** | - | 2 | - | - | 1 | - | - | 2 |
| **3** | 1 | 1 | 2 | 3 | 1 | - | - | 1 |
| **4** | 1 | 1 | - | 5 | 1 | 1 | 2 | - |
| **5** | 3 | 1 | - | 1 | - | 1 | 1 | - |
| **6** | 1 | 1 | 1 | - | - | 1 | 2 | 4 |
| **7** | 1 | 1 | 2 | 1 | - | 1 | 9 | - |
| **8** | 5 | 1 | 4 | 1 | 1 | - | 1 | 1 |
| **9** | - | 1 | 1 | 1 | 1 | - | - | 1 |
| **10** | 1 | 1 | 1 | 1 | 1 | - | 1 | - |
| **11** | 4 | 5 | - | 1 | 2 | 1 | - | - |
| **12** | 3 | - | - | 1 | 1 | 4 | 4 | - |
| **13** | 1 | 1 | - | 1 | 2 | - | 6 | - |
| **14** | 1 | 4 | - | 2 | 1 | 2 | - | 2 |
| **15** | 1 | 1 | 1 | 3 | - | 1 | 8 | - |
| **16** | 1 | - | 2 | 1 | 1 | 2 | - | 1 |
| **17** | 1 | 1 | - | 1 | 1 | 1 | 1 | 1 |
| **18** | 2 | 1 | 1 | 1 | - | - | 1 | 1 |
| **19** | 1 | - | - | 1 | 1 | - | 3 | - |
| **20** | 1 | 2 | 4 | 1 | 3 | 1 | 5 | - |
| **21** | 1 | 1 | 2 | 1 | 1 | - | 5 | 1 |
| **22** | - | 1 | - | 2 | - | - | 1 | - |
| **23** | 1 | 1 | - | 2 | 1 | 1 | 7 | 1 |
| **24** | 1 | 4 | 1 | 2 | - | 2 | - | 2 |
| **25** | 4 | 1 | - | 1 | 1 | 1 | - | 1 |
| **26** | 1 | 2 | 1 | 1 | - | 1 | 1 | - |
| **27** | 1 | 1 | - | - | 1 | 1 | - | 1 |
| **28** | 1 | 3 | 1 | 1 | 1 | 1 | - | 3 |
| **29** | 1 | 1 | 1 | 1 | - | 1 | - | 1 |
| **30** | 1 | 1 | - | 1 | 2 | - | - | - |
| **31** | 3 | 1 | - | 5 | 1 | 1 | - | 2 |
| **32** | 2 | 1 | - | 1 | 2 | 1 | - | - |
| **33** | 1 | 2 | 3 | 2 | 4 | 4 | - | 1 |
| **34** | - | 1 | - | 1 | 2 | 2 | - | 2 |

| | | | | | | | | |
|---|---|---|---|---|---|---|---|---|
| **35** | 1 | 1 | 1 | 1 | - | - | - | 1 |
| **36** | 8 | 1 | 1 | 1 | 1 | - | - | 1 |
| **37** | 1 | 2 | 1 | 1 | 1 | - | - | 1 |
| **38** | 2 | 1 | 1 | 4 | 1 | 1 | - | 1 |
| **39** | 2 | 1 | 1 | 1 | 2 | - | - | 1 |
| **40** | 1 | - | - | 1 | 1 | 2 | - | 1 |
| **41** | 1 | 1 | 1 | 1 | 2 | 1 | - | 1 |
| **42** | 2 | 1 | 1 | 1 | 1 | - | - | 1 |
| **43** | 1 | 1 | - | - | 1 | 1 | - | 1 |
| **44** | 1 | 1 | 1 | 1 | 1 | 1 | - | 1 |
| **45** | - | 1 | 1 | 1 | 1 | 1 | - | - |
| **46** | - | 2 | 2 | 1 | 4 | 1 | - | 1 |
| **47** | - | 1 | 1 | 1 | 1 | 1 | - | - |
| **48** | - | 2 | 2 | 1 | 1 | 1 | - | 1 |
| **49** | - | 1 | 1 | 1 | - | 1 | - | 1 |
| **50** | - | 1 | 2 | 2 | 1 | 1 | - | 1 |
| **51** | - | 2 | 1 | 1 | 5 | 1 | - | 1 |
| **52** | - | 1 | 3 | 1 | - | 1 | - | 4 |
| **53** | - | 1 | 1 | - | 1 | - | - | 1 |
| **54** | - | - | 1 | - | - | 1 | - | 1 |
| **55** | - | - | 1 | - | - | 4 | - | 1 |
| **56** | - | - | 1 | - | 2 | 1 | - | - |
| **57** | - | - | 1 | - | 2 | 2 | - | 1 |
| **58** | - | - | 1 | - | 1 | 1 | - | 3 |
| **59** | - | - | 1 | - | 1 | 3 | - | 3 |
| **60** | - | - | 1 | - | 1 | - | - | 1 |
| **61** | - | - | 1 | - | 1 | 1 | - | 1 |
| **62** | - | - | - | - | 1 | - | - | 2 |
| **63** | - | - | - | - | - | 2 | - | 1 |
| **64** | - | - | - | - | - | 3 | - | 3 |
| **65** | - | - | - | - | - | 1 | - | - |
| **66** | - | - | - | - | - | 1 | - | - |
| **67** | - | - | - | - | - | 1 | - | 1 |
| **68** | - | - | 1 | - | - | - | - | - |
| **71** | - | - | 4 | - | - | - | - | - |
| **72** | - | - | 1 | - | - | - | - | - |

| | | | | | | | | |
|---|---|---|---|---|---|---|---|---|
| **73** | - | - | 1 | - | - | - | - | - |
| **74** | - | - | 1 | - | - | - | - | - |
| **75** | - | - | 1 | - | - | - | - | - |
| **82** | - | - | 1 | - | - | - | - | - |
| **Unique** | 40 | 48 | 48 | 47 | 47 | 48 | 18 | 48 |
